# The Role of Endoscopic Ultrasound for Esophageal Varices

**DOI:** 10.3390/diagnostics10121007

**Published:** 2020-11-25

**Authors:** Kazunori Nagashima, Atsushi Irisawa, Keiichi Tominaga, Ken Kashima, Yasuhito Kunogi, Takahito Minaguchi, Naoya Izawa, Akira Yamamiya, Akane Yamabe, Koki Hoshi, Kenichi Goda, Makoto Iijima

**Affiliations:** Department of Gastroenterology, Dokkyo Medical University School of Medicine, 880 Kitakobayashi Mibu, Tochigi 321-0293, Japan; n-kazu@dokkyomed.ac.jp (K.N.); tominaga@dokkyomed.ac.jp (K.T.); ken-k@dokkyomed.ac.jp (K.K.); ykunogi@dokkyomed.ac.jp (Y.K.); takahito@dokkyomed.ac.jp (T.M.); izawanao@dokkyomed.ac.jp (N.I.); akira-y@dokkyomed.ac.jp (A.Y.); yamabe@dokkyomed.ac.jp (A.Y.); hoshi@dokkyomed.ac.jp (K.H.); goda@dokkyomed.ac.jp (K.G.); mkiijima@dokkyomed.ac.jp (M.I.)

**Keywords:** esophageal varices, endoscopic ultrasound, hemodynamics, endoscopic injection sclerotherapy, endoscopic variceal ligation

## Abstract

Esophageal varices are caused by the development of collateral circulation in the esophagus as a result of portal hypertension. It is important to administer appropriate preventive treatment because bleeding varices can be fatal. Esophageal varices have complex and diverse hemodynamics, and there are various variations for each case. Endoscopic ultrasound (EUS) can estimate the hemodynamics of each case. Therefore, observation by EUS in esophageal varices provides useful information, such as safe and effective treatment selection, prediction of recurrence, and appropriate follow-up after treatment. Although treatment for the esophagogastric varices can be performed without EUS imaging, understanding the local hemodynamics of the varices using EUS prior to treatment will lead to more safe and effective treatment. EUS observation is an indispensable tool for thorough variceal care.

## 1. Introduction

Transabdominal ultrasound (US), computed tomography (CT), and magnetic resonance imaging (MRI) are established diagnostic tools for abdominal diseases. Despite their widespread use, these techniques have limitations. In contrast, endoscopic ultrasound (EUS) complements these modalities, and has been proven useful for evaluating the mediastinum, pancreas, biliary tract, gastrointestinal tract, liver, and adrenal glands [1,2,3,4].

Esophageal varices are caused by the development of collateral circulation in the esophagus as a result of portal hypertension. It is important to administer appropriate preventive treatment because bleeding varices can be fatal. Varices found in the esophageal lumen are just the tip of the iceberg. To prevent the occurrence of varices, it is important to evaluate and understand the underlying hemodynamics associated with portal hypertension. In the actual preventive treatment of varices, effective treatment methods and factors associated with recurrence after treatment differ according to hemodynamics [5,6,7,8]. As in the case of other organs, contrast-enhanced CT (3D construction) and contrast-enhanced MRI are useful for determining portal vein hemodynamics in cases of portal hypertension. However, while these modalities are useful for understanding the state of the portal vein overall, they are insufficient for determining localized detailed hemodynamics of esophageal varices. EUS, however, enables observation near esophageal varices and helps to understand the localized detailed hemodynamics of esophageal varices [9,10]. This suggests that useful information can be obtained for the selection of treatment suited to the hemodynamics of each individual varix and for the prediction of recurrence [5,6,7,8]. Furthermore, EUS is considered highly useful for determining the treatment response and can easily evaluate the presence or absence of residual blood flow that is difficult to determine by normal endoscopic observation [5]. In this review article, we conducted a review of the literature of the effectiveness of EUS diagnosis and its role in the treatment of esophageal varices.

## 2. Hemodynamics of Esophageal Varices

Increased intrahepatic vascular resistance and the volume of portal vein blood flow causes increased portal vein pressure, which results in a countercurrent phenomenon from the portal vein, thus resulting in the formation of esophageal varices. The blood vessels involved in the formation of esophageal varices include feeding veins, such as the left gastric vein (LGV), posterior gastric vein, and short gastric vein, as well as vessels with a bamboo blind-like pattern, paraesophageal veins, and perforating veins. The collateral pathway of the portal vein that develops in relation to esophageal varices is primarily the pathway communicating the portal vein and azygos vein. A Japanese study of percutaneous trans hepatic portography targeting 65 patients with liver cirrhosis accompanied with esophageal varices revealed that, in all patients, the primary pathway that was feeding the esophageal varices was the LGV [11].

**Figure 1:**
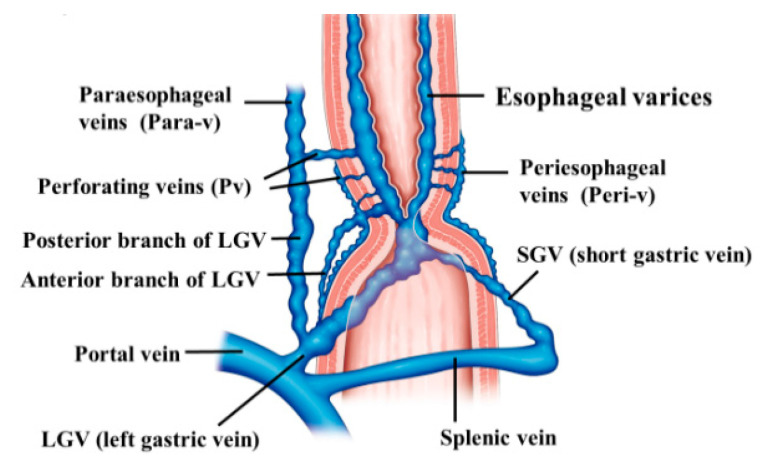
Diagram of the hemodynamics of esophageal varices.

In most cases of esophageal varices, the esophageal varices form via bamboo blind-like vessels from the anterior branch of the LGV, and drain blood into the azygos vein (Figure 1). By contrast, in some cases, the posterior branch of the LGV communicates with the paraesophageal veins without passing the bamboo blind-like vessels, and the varices are fed from the paraesophageal veins via the veins perforating the esophageal wall. The morphology and hemodynamics of the LGV in patients with portal hypertension were studied to evaluate factors affecting the development of esophageal varices [12]. It was shown that the diameter of the LGV trunk increases with increasing variceal size. This study also demonstrated the branching pattern of the LGV and its relationship with collateral channels. This branching pattern was found to be closely associated with the development of esophageal varices because dominance of the anterior branch may be responsible for directing LGV blood flow toward varices at the level of the proximal stomach [12]. However, it has been pointed out that inflow of the LGV of the upper third of the stomach also contributes to the onset and development of varices. Kiyono et al. [13] noted that the hemodynamics in the left gastric artery may act as an initiator of variceal formation, and that the LGV contributes to the onset of esophageal varices. On angiography of the LGV, there was a difference observed in the mean detection time for recurrent esophageal varices between patients with recurrent varices (7.0 s) and patients without recurrence (5.6 s). Thus, various hemodynamics are involved in esophageal varices, making it important to understand the underlying pathology for treatment.

## 3. EUS Procedure for the Diagnosis of Esophageal Varices

**Figure 2:**
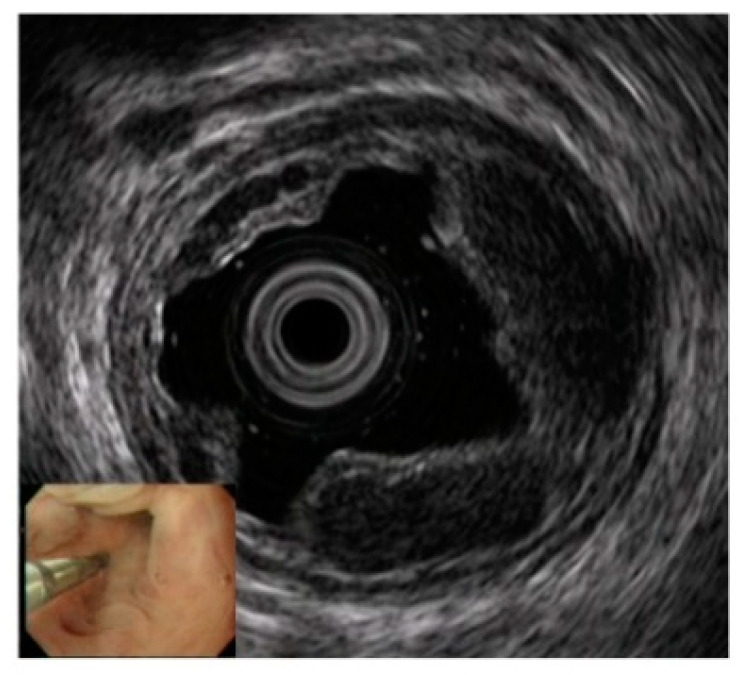
Observation of esophageal varices using a thin probe (20 MHz UMP, Olympus Co., Tokyo, Japan). Even narrow blood vessels can be observed.

**Figure 3:**
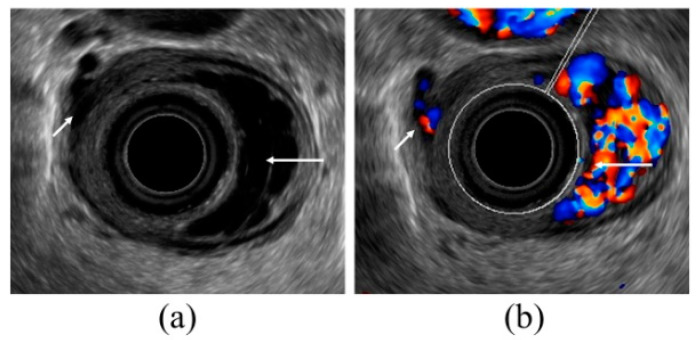
Observation of esophageal varices using a radial-arrayed echoendoscope (FUJIFILM, EG-580UR). (**a**) Observation in B-mode. Perforating veins (short arrow) and esophageal varix (long arrow) are observed. (**b**) Color Doppler observation. The esophageal varix (long arrow) is seen as a flow of mixed red and blue signals. Furthermore, the perforating vein (short arrow) is primarily presented in blue, and this site acts as the outflow passage from esophageal varices.

For EUS observation of varices, a thin probe (20 MHz ultrasonic mini probe (UMP)) and a specialized EUS device (electronic radial-arrayed EUS) are used. When conducting detailed observation of the morphology of varices and localized hemodynamics surrounding varices, the 20 MHz UMP with high resolution should be used. Using this type of device enables observation of the 1–2 mm blood vessel group that is difficult to observe in detail on conventional EUS devices (Figure 2). When performing electronic radial-arrayed EUS, it is difficult to achieve detailed esophageal intramural and extramural evaluation, which is possible with UMP. However, the color Doppler function enables identification of the direction of the blood flow of perforating veins communicating between the esophageal varices and extramural blood vessels, and it also aids in understanding the hemodynamics surrounding the esophageal varices in greater detail [14,15] (Figure 3). Perforating veins with blood flow into esophageal varices may remain after treatment and contribute to recurrence, which suggests the importance of eliminating these at the time of treatment. Ideally, these two types of devices will be used properly in accordance with the requirements of each case. Normally, observation is performed using conventional UMP, and if a large number of perforating veins are markedly observed, electronic radial-arrayed EUS is used to observe the perforating veins, including the blood flow direction.

### 3.1. UMP Endoscopy Technique

Essentially, the entire circumferences of extramural blood vessels of the esophagus and stomach are observed extending the entire area from the upper third of the stomach to the lower esophagus using the de-aired water filling method. In particular, for observation using UMP, observation is performed using a scope with an automatic water supply function, or upon attaching a water supply device to the opening of normal endoscopic forceps, and while filling the esophageal lumen with de-aired water. Observation of the mucosa and esophageal submucosal tumors using the jelly-filled method, whereby the esophagus is filled with medical jelly, has also been reported [16,17], and can be applied for esophageal varices. With this technique, highly viscous jelly remains in the esophagus for a relatively long time, thereby causing the esophageal wall to extend and making it difficult for bubbles to enter, which is believed to enable clearer images to be obtained compared to flowing water. There are no reports of the impact on objective evaluations and comparing the effectiveness of the de-aired water filling method against the jelly-filled method. No consensus has been drawn as to which is more useful; however, in actual EUS observation, the de-aired water filling method is generally used because of its simplicity. We believe that further examination to compare the effectiveness is needed. The specific procedure first involves injecting approximately 300–400 mL of de-aired water while projecting the UMP approximately 15 mm from the scope tip. Following this, observation can be commenced while injecting de-aired water into the esophageal lumen (when using lubricating jelly, commencing after injecting 20–30 mL into the esophageal cavity). Observation is performed while gently pulling out the scope from directly beneath the cardia. Furthermore, due care should be paid to the fact that when using the automatic water supply function, strong water flow can cause the formation of bubbles, thereby impairing observation [18]. In addition, it should be noted that the infusion of a lot of water into the esophagus can cause aspiration pneumonia. Moreover, with the jelly-filled method, there is concern about how resistance to jelly injection affects post-observation procedures (particularly endoscopic variceal ligation (EVL)).

### 3.2. Observation of Esophageal Varices Using the Endoscopic Technique of Electronic Radial-Arrayed EUS

In recent years, the forward-viewing type radial-arrayed echoendoscope (FUJIFILM; EG-580UR, PENTAX Medical; EG-3670URK) has become commercially available (Figure 3). This technique enables easy EUS observation following conventional varix observation. Furthermore, even with liner-arrayed (convex) EUS, varices can be observed; however, for observation of esophageal varices, inadequate observation can be obtained in that full-circumferential observation must be performed. With observation using electronic radial-arrayed EUS, intramural and extramural blood vessels of the esophagus and stomach can be observed in a full-circumferential manner, extending the entire region from the upper third of the stomach to the lower esophagus. The observation of extramural blood vessels is performed upon inflating the tip balloon. However, when observing esophageal varices in the esophagus, compression caused by the balloon makes the intended varix observation impossible, and the de-aired water filling method is therefore used per UMP. Because the color Doppler function can be used, more information can be obtained compared to UMP [19,20]. Sato et al. [14] reported that among 62 patients, color Doppler images of esophageal varices and of the paraesophageal veins could be obtained using EUS in all patients. Furthermore, it has been reported that by using Levovist^®^ (Schering AG, Berlin, Germany), which is a microbubble contrast agent for image diagnosis by intravenous ultrasonography, the diagnostic quality is improved and images are clearer than EUS images.

## 4. The Role of EUS for Esophageal Varices

The significance of observing esophageal varices by EUS lies in determining the risk of bleeding, preoperative evaluation for safe and effective treatment, predicting recurrence, and determining the therapeutic response.

### 4.1. Evaluation of the Risk Factors of Variceal Bleeding

With regard to the risk of variceal bleeding, red color signs are considered extremely important endoscopic findings; among these, hematocystic spots are particularly important endoscopic findings. Schiano et al. [21] reported that EUS can identify hematocystic spots on the surface of esophageal varices, the presence of which is strongly associated with a high risk of esophageal variceal rupture. These appear as saccular aneurysms, similar to projections on the variceal surface, as observed using high-resolution endoluminal sonography. It has also been reported that the larger the varix, the greater the risk of bleeding; however, the varix size can be objectively evaluated using EUS and the risk of bleeding can be predicted [22].

### 4.2. Evaluation of the Hemodynamics of Esophageal Varies

**Figure 4:**
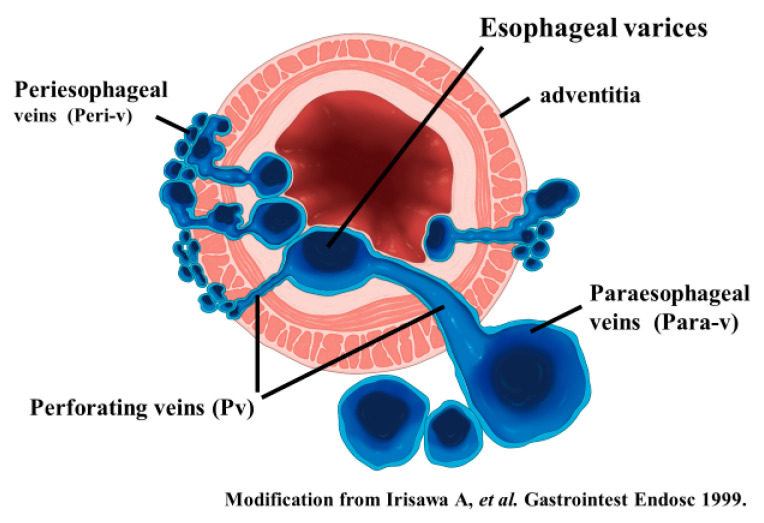
Diagram of UMP observation of esophageal varices. UMP can reveal collateral vessels inside and outside the esophageal wall in detail. The esophageal collateral veins (ECVs) are divided into two types, peri- and para-ECVs. In addition, perforating veins between esophageal varices and ECVs are also seen.

**Figure 5:**
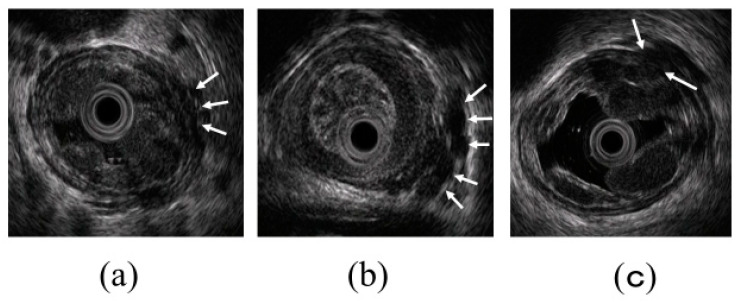
Intramural and extramural observation items of the esophagus. (**a**) Peri-ECVs. Many blood vessels of small diameter are observed adjacent to the esophageal adventitia (arrow). The existence of these vessels makes the margin of the musculus longitudinalis externus appear unclear. (**b**) Para-ECVs. Blood vessels with a relatively large diameter located distant to the esophageal adventitia (arrow). (**c**) Veins perforating the esophageal wall. Perforating veins communicating between the esophageal varices and extramural ECVs (arrow).

To administer safe preventive treatment of varices, it is indispensable to evaluate the underlying hemodynamics, and, in particular, preoperative evaluation by EUS is considered important [23,24,25,26]. In conventional endoscopic observation, only esophageal varices within the esophageal lumen can be observed, and the extramural collateral pathways involved in the development of the varices cannot be observed. Endoscopic treatment of esophageal varices involves localized inhibition of blood flow. However, to execute such treatment safely and effectively, it is important to not only be able to see the esophageal varices by endoscopy, but also to understand the underlying structure, i.e., the hemodynamics, including the feeding vessels. As noted above, contrast-enhanced CT and contrast-enhanced MRI are useful for identifying the general portal vein hemodynamics. However, for localized observation of esophageal varices (such as vessels with a bamboo blind-like pattern, paraesophageal veins, and perforating veins), the use of EUS is needed. Observation items to evaluate hemodynamics of localized esophageal varices include the peri-esophageal collateral veins (peri-ECVs), paraesophageal collateral veins (para-ECVs), and perforating vein (PV) [24] (Figure 4). The peri-ECVs are a group of blood vessels with a small diameter observed adjacent to the esophageal adventitia, and in developing cases, the margin of the esophageal muscularis propria (musculus longitudinalis externus) appears shaggy (Figure 5a). Para-ECVs, however, are a group of blood vessels with a relatively large diameter found clearly apart from the esophageal wall (Figure 5b). Para-ECVs have also been studied, and a histopathological causal relationship has been demonstrated by the existence of both peri-, and para-ECVs [27]. Furthermore, these ECVs often communicate with esophageal varices via PV, and such relationships can be observed in detail on EUS [14,15,24,27] (Figure 5c). According to studies of the blood flow direction of the PV using color Doppler EUS, it was found that most PV in the lower esophagus served as inflow pathways [14,15]. Thus, peri-ECVs and para-ECVs with PVs serve as feeding pathways from the lateral esophagus. Figure 1 presents esophageal varices and the surrounding vascular structure seen in a patient with portal hypertension [24]. Moreover, Hino et al. [12] reported that they were able to observe the LGV using color Doppler EUS (CD-EUS), which is the original feeding pathway of esophageal varices. While the purpose of the UMP is thorough observation of localized hemodynamics of esophageal varices, using specialized EUS devices enables the observation of overall portal vein hemodynamics.

### 4.3. The Interpretation of the Esophageal Variceal Hemodynamics by EUS

As mentioned above, ECVs and PV observed near esophageal varices can become feeding pathways for esophageal varices. Thus, when the PV and ECVs communicating with esophageal varices via PV remain, it is inferred that they greatly contribute to variceal recurrence [25,28,29,30,31]. Irisawa et al. [25] divided the development of peri-ECVs into mild and severe, and examined how such development was involved in recurrence after endoscopic injection sclerotherapy (EIS). Furthermore, with regard to PV, they examined how the diameter of such blood vessels contributed to recurrence. According to their results, EUS findings included a significantly (*p* < 0.001) higher incidence of severe type peri-esophageal collateral veins, a significantly larger number of perforating veins (*p* < 0.001), and perforating veins with a significantly larger diameter (*p* < 0.001) compared with patients without recurrence (8 of 10, 80% vs. 2 of 28, 7.1%; 1.30 vs. 0.21; 2.00 vs. 0.32 mm, respectively). Furthermore, Masalaite et al. [32] examined factors involved in recurrence following EVL using EUS, and Irisawa et al. reported that univariate logistic regression analysis showed that severe peri-ECV (*p* < 0.001), multiple peri-ECV (*p* < 0.001), and the presence of perforating veins (*p* < 0.014) were statistically significantly related to the variceal recurrence after EBL. They also reported that the multivariate logistic regression model revealed that only severe peri-ECV (odds ratio (OR) = 24.39; 95% confidence interval (CI): 2.34–253.78) and multiple peri-ECV (OR = 24.39; 95% CI: 2.34–253.78) remained as independent prognostic factors for variceal recurrence. However, many opinions exist regarding para-ECVs, and the role of para-ECVs remains controversial. The thickness of the PV diameter and communication with PV have been reported to contribute to bleeding and recurrence [33,34,35]. Leung et al. [34] reported that recurrent submucosal esophageal varices were detected in 24 patients, including 13 patients (93%) with large PEVs and 11 patients (46%) with no or small PEVs (*p* = 0.0019). Recurrent bleeding occurred in six patients (43%) with large PEVs and in three patients (12%) with small PEVs (*p* = 0.044), and they pointed out that para-ECVs that developed were observed by EUS, contributing to recurrence. Similarly, Carneiro et al. [33] reported that larger paraesophageal varices predicted variceal recurrence in both evaluation periods. Diameters of paraesophageal varices that best correlated with variceal recurrence were 6.3 mm before EBL (52.9% sensitivity, 92.3% specificity, and 0.749 area under the receiver operating characteristic curve (AUROC)) and 4 mm after EBL (70.6% sensitivity, 84.6% specificity, and 0.801 AUROC). However, it has also been reported that para-ECVs do not necessarily contribute to recurrence. Irisawa et al. [36] examined the involvement of para-ECVs in recurrence following EIS using EUS and liver scintigraphy, and reported that the para-ECVs are collaterals, which reflect the portal blood flow after endoscopic sclerotherapy, and that the para-ECVs without perforating veins were considered important collaterals after treatment. As a result, it was found that while they contribute to the presence of PV, para-ECVs were found to be blood vessels with an important buffer action on portal vein pressure. In fact, Shibukawa et al. [37] examined the involvement of the PV on variceal recurrence using EUS, with the esophagogastric junction blood vessels showing involvement of the LGV and PV divided into four groups (group A: perforating veins (+) and vessels at esophagogastric junction (+); group B: perforating veins (+) and vessels at esophagogastric junction (−); group C: perforating veins (−) and vessels at esophagogastric junction (+); and group D: perforating veins (−) and vessels at esophagogastric junction (−)), and they found that, although there was no significant difference between group A or C and B, there was a significant difference between group A or B and D. Therefore, the authors concluded that the perforating veins are highly associated with variceal recurrence after sclerotherapy, even if perforating veins are independent.

Furthermore, it has been previously pointed out that the feeding pathway that directly develops from the portal vein (such as the LGV) contributes to variceal recurrence. Toyonaga et al. [38] demonstrated that the prevalence of the “pipeline” form of variceal feeding pattern (a large dilated LGV running up the esophagus) was higher in patients with resistant varices than in those with non-resistant varices (100 vs. 3%, respectively; *p* < 0.01), and the diameter of the LGV was larger in patients with resistant varices than in those with non-resistant varices (12.4 ± 2.0 vs. 7.8 ± 2.3 mm, respectively; *p* < 0.01). Supported by such studies, the LGV has been examined using EUS. Hino et al. [12,23] reported that either a high hepatofugal flow velocity in the LGV or an anterior branch dominant pattern seen under CD-EUS was a possible contributing factor for variceal recurrence after endoscopic treatment. The same group recently published a larger study of 68 patients [18] treated for moderate or large esophageal varices with variceal ligation and sclerotherapy after CD-EUS. The authors concluded that their results suggest that patients showing anterior branch dominance and rapid hepatofugal flow velocity in the LGV on CD-EUS examination are at high risk of early recurrence of esophageal varices.

### 4.4. Evaluation of the Therapeutic Effect

Observation by EUS following completion of variceal treatment provides useful information about the need for additional treatment and follow-up observation. As a result of thrombus formation in varices after treatment, the hypoechoic signals disappear and change to hyperechoic signals on ultrasound images. However, when hypoechoic lumen is observed in hyperechoic images, it can be understood to mean that the thrombus formation is incomplete. Pontes et al. [39] examined variceal changes after treatment and reported that EUS showed insufficient variceal thrombosis in six (17%) patients who appeared to have variceal eradication at endoscopy after treatment. EUS was also superior to endoscopy for diagnosing gastric varices, and showed patent vessels in 26 (74%) out of 35 patients, indicating the importance of EUS observation after treatment. Similarly, Suzuki et al. [40] observed the vascular structure at the esophagogastric junction using EUS after the endoscopic treatment of varices and examined the relationship with recurrence. After variceal eradication, a US miniature probe showed intramural vessels in the cardia that were classified as follows: grade I, a few vessels (19 patients, 46%); grade II, uniformly scattered vessels (11, 27%); and grade III, abundant vessels resembling a honeycomb (11, 27%). They noted that as the sonographic grade increased, the rate of variceal recurrence increased, and as the venographic grade of staining in the distal esophagus increased, the esophageal wall became thicker and the sonographic grade at the cardia increased, which was found to be useful for the evaluation of treatment and for the prediction of recurrence.

Furthermore, as mentioned above, it has been reported that peri-ECVs and para-ECVs, as well as the PV communicating between these vessels and esophageal varices, contribute to recurrence; however, determining whether or not these blood vessel groups have disappeared after endoscopic treatment is also important. If the disappearance of these blood vessel groups could be confirmed by EUS, we believe this indicates that sufficient therapeutic outcomes have been obtained.

Moreover, if the LGV, which is the direct feeding pathway from the portal vein, remains, then it greatly contributes to recurrence. Using CD-EUS enables the evaluation of the presence or absence of residual blood flow of the LGV after treatment [23]. When performing EIS in particular, observation using EUS to determine the presence or absence of thrombosis of the feeding pathway is of great significance.

## 5. Application to Treatment for Esophageal Varices

The question of how to apply information obtained by EUS to treatment is an important point. Selecting treatment with a low recurrence rate and that leads to improved patient quality of life (QOL) based on information observed by EUS is important.

There are two types of endoscopic preventive treatment methods for esophageal varices, i.e., EIS and EVL [41,42]. EIS is a treatment method involving embolization of the feeding pathway by injecting a sclerosing agent (ethanolamine oleate) into the varices. It is a therapeutic method of inhibiting hemodynamics, and has been found to keep the rate of recurrence low. However, because of the complexity of the procedure and that hemodynamics need to be understood, it can be called a highly specialized treatment. In contrast, EVL is a technique of polypoid ligation of varices, and a therapeutic method of physically blocking the variceal blood flow and promoting fibrosis. Owing to the technical simplicity, the technique has gained widespread popularity [42]. However, with EVL alone, which is a localized treatment of varices, the effect of feeding pathway embolization cannot be expected, and the high rate of recurrence is considered a problem [43,44]. Understanding the hemodynamics of esophageal varices by EUS is useful for the selection of the treatment method. In this respect, Nakamura et al. [33] conducted a remarkably interesting study using three-dimensional EUS (3D-EUS). The images on EUS were divided into four patterns (the pattern of variceal blood flow detected on 3-D images was classified into type 1 (cardial inflow without paraesophageal veins), type 2 (cardial inflow with paraesophageal veins), type 3 (azygos-perforating pattern) and type 4 (complex pattern)), and examined the relationship with recurrence following EIS or EVL. Based on the 3D-EUS data, 41 patients (46.1%) were classified as type 1, 12 (13.5%) as type 2, seven (7.9%) as type 3, and 29 patients (32.6%) as type 4. The cumulative recurrence-free probability at 24 months after treatment was 28.9% for ligation versus 71.1% for sclerotherapy (*p* < 0.05) in type 1, whereas the respective probabilities were 72.9% vs. 50.0% (NS) for type 2 varices, 100% vs. 100% (NS) for type 3 varices, and 61.9% vs. 64.8% (NS) for type 4 varices. Based on these results, it was concluded that ligation is indicated for patients who have collaterals, such as paraesophageal veins running parallel to the varices, as the blood flow can be diverted to these blood vessels and controlled by localized ligation. Furthermore, it is difficult to block PV by ligation, and it is preferable to treat the PV with sclerotherapy if it has a large diameter. As mentioned above, as the PV is an independent factor that contributes to recurrence, Shibukawa et al. noted the importance of developing an appropriate treatment strategy based on EUS findings. Nagamine et al. [45], in order to decrease variceal recurrence, conducted a pilot study of a “modified” EVL technique in conjunction with a UMP. Esophageal varices were imaged by UMP followed by intensive EVL therapy. EVL was repeated every second week until varices showed complete eradication or marked reduction and the red color sign became negative. The reported intermediate term (12 – 24 months) outcome of patients treated by this technique was good. Based on EUS findings, the development of such treatment is also considered important. EUS-guided sclerotherapy targeting the PV has also been reported. Lahoti et al. [46] conducted EUS sclerotherapy using Varijet (2.5 mm catheter) injector needles and sodium morrhuate directed at the perforating vessels until the flow was completely impeded (2–4 mL per injection site), and reported that dynamic EUS-guided sclerotherapy with color flow Doppler may be safely and effectively used for the treatment of esophageal varices. Therefore, if the blood vessels involved in recurrence could be identified in advance by EUS, this would be highly effective for treatment, such as selective treatment of the blood vessels concerned, and for the EUS observation of esophageal varices.

Other reports have indicated that the recurrence inhibitory effect of propranolol, which reduces portal vein pressure, can also be evaluated by EUS. Liao et al. [47] examined the relation between EV recurrence after treatment by ligation and volumetric change of PEV in patients undergoing EVL with and without propranolol; they found that propranolol may reduce both the EV recurrence rate and volume of PEV in patients achieving endoscopic eradication. Therefore, EUS provides useful information to help determine the effects of pharmacotherapy.

## 6. Conclusions

Esophageal varices present various complex hemodynamics and as many variations according to each patient; however, observation by EUS including UMP enables the hemodynamics of each patient to be roughly estimated. The observation of esophageal varices by EUS enables obtaining useful information, such as that for the selection of safe and effective treatment methods, predicting prognosis, and for appropriate follow-up observation after treatment. In particular, the selection of appropriate treatment methods according to the results of the observation of blood vessel groups surrounding esophageal varices (peri-ECVs, para-ECVs, and PV), which are believed to contribute to recurrence after treatment, promotes good QOL for patients with esophageal varices. However, EUS has some limitations, such as operator dependence (technical and cognitive skills) and a long learning curve. Therefore, in order to fully apply the usefulness of EUS to clinical practice, it is necessary to standardize EUS procedures and image interpretation, and develop the training tools. Furthermore, support for diagnostic imaging by artificial intelligence may be indispensable for future development [48,49,50]. We believe that EUS observation is an indispensable tool for thorough variceal care.
